# RELA-induced MiR-21 Exerts Oncogenic Effects on PDAC via Targeting of ARHGAP24

**DOI:** 10.7150/jca.73690

**Published:** 2022-06-21

**Authors:** Lanting Yu, Jiawei Lu, Haoran Xie, Jianbo Ni, Sumin Chen, Qiuyan Zhao, Ni Xie, Lungen Lu, Xingpeng Wang, Baiwen Li

**Affiliations:** 1 Department of Gastroenterology, Shanghai General Hospital, Shanghai Jiao Tong University School of Medicine, Shanghai 201620, China.; 2 Shanghai Key Laboratory of Pancreatic Diseases, Shanghai General Hospital, Shanghai Jiao Tong University School of Medicine, Shanghai 201620, China.; 3 Department of Gastroenterology, Tongren Hospital, Shanghai Jiao Tong University School of Medicine, Shanghai 200336, China.

**Keywords:** Pancreatic ductal adenocarcinoma, ARHGAP24, RELA, microRNA, Transcription factor

## Abstract

Inflammation is one of the inducing factors of pancreatic ductal adenocarcinoma (PDAC), and microRNAs have been confirmed to be involved in the occurrence and development of PDAC. However, whether RELA, an inflammatory regulator, is involved in the regulation of PDAC by miRNA remains to be further studied. In the present study miR-21 was characterized and its upstream regulatory mechanism was investigated, as well as its functional effects and target genes in pancreatic ductal adenocarcinoma (PDAC). *In situ* hybridization analysis confirmed increased miR-21 expression levels in PDAC tissues. The results of the chromatin immunoprecipitation and dual-luciferase reporter assays demonstrated that transcription factor RELA modulated miR-21 transcription in the PDAC, PANC-1 and MIA PaCa-2 cell lines. Subsequently, a cell viability assay, EdU staining assay and flow cytometry analysis, demonstrated that miR-21 promoted cell proliferation and cell cycle progression, but inhibited cell apoptosis *in vitro*. Furthermore, a xenograft assay demonstrated that miR-21 accelerated tumor growth *in vivo*. Mechanistically, miR-21 directly regulated the expression of Rho GTPase activating protein 24 (ARHGAP24), which was indicated to be a tumor suppressor gene. Moreover, both miR-21 and ARHGAP24 were strongly associated with clinical features and may therefore serve as valuable biomarkers in PDAC prognosis.

## Introduction

Pancreatic ductal adenocarcinoma (PDAC) is a highly malignant type of cancer. PDAC has a high mortality rate due to its late detection and lack of effective treatment. It is therefore predicted to be the second leading cause of cancer-related deaths worldwide by 2030 1. Consequently, new insights into the mechanisms driving the initiation and development of PDAC are needed.

It has been founded that aberrant RNA expression is involved in the malignant progression of PDAC. MicroRNAs (miRNAs/miRs) are small endogenous noncoding RNAs that are involved in a wide range of cellular processes 2, 3. Evidence suggests that targeting oncogenic miRNAs may serve as a promising therapeutic approach. Liu *et al.* 4 reported that in head and neck cancer, sophocarpine can be used to target miR-21, exhibiting a promising therapeutic effect. Previous studies have also reported that a combination of chemotherapeutics and miR-21 inhibitors reverse drug resistance in colon cancer. Furthermore, miRNAs have the potential to act as prognostic indicators 5, 6.The pathological and physiological functions of miR-21 have been intensively studied, while the mechanism underlying its abnormal expression is less explored, especially with regards to the upstream transcriptional regulation of miR-21. Herein, to further understand the molecular mechanisms of miR-21 in PDAC and to identify potential therapeutic targets, we focused on its transcriptional regulation and downstream mechanisms.

RELA proto-oncogene NF-κB subunit (RELA), also named as p65, belongs to the nuclear factor κB (NF-κB) transcription factor family. It has been demonstrated that RELA regulated the transcription of genes involving in cell proliferation, apoptosis and metastasis, such as cyclin D1, PCNA and Bcl-2^7^, which entitled RELA to an oncogenic role in breast cancer, hepatocellular cancer and other types of cancer^8^, ^9^. Mechanically, it can regulate the expression of non-coding RNAs. In PDAC, RELA was overexpressed and was found to participate in the cell proliferation and migration of PDAC by regulating NEAT1 (a non-coding RNA). However, the relationship between RELA and mir-21 remains unclear, as well as the interaction between RELA and miR-21 could affect the biological behavior of PDAC cells.

Rho GTPase activating protein 24 (ARHGAP24) is a Rac-specific Rho GTPase activating protein (Rho GAP), a negative regulator of Rho GTPase activity. It has shown its regulating role in cell proliferation, invasion and metastasis 10, 11. Recent studies have indicated that ARHGAP24 suppressed cell proliferation and cycle progression, and promoted apoptosis in lung cancer. It can also inhibit the invasion and metastasis of triple-negative breast cancer 12, 13. So far, whether ARHGAP24 is involved in the development of PDAC has remained uninvestigated. Through miRNA target prediction algorithms, ARHGAP24 was found to be a potential candidate of miR-21. Considering PDAC harbors a high frequency of genetic alterations, the relationship and function of these target genes need to be further studied.

In this study, we found that RELA induces the high expression of miR-21 in PDAC. Additionally, ARHGAP24 was identified as the target of miR-21 and mediated the effects of miR-21 in PDAC cells. Furthermore, we demonstrated that both miR-21 and ARHGAP24 are correlated with PDAC clinical features. Taken together, these findings may provide novel diagnostic and therapeutic targets for PDAC.

## Materials and methods

### Online database analysis

The prognostic value of miR-21 expression and ARHGAP24 mRNA expression was evaluated using an online database, Kaplan-Meier Plotter. To analyze the overall survival (OS) and relapse free survival (RFS) of patients with PDAC, patient samples were split into two groups by median expression (high vs. low expression) and assessed using a Kaplan-Meier survival plot, via the hazard ratio with 95% confidence intervals and log-rank P-value. The Cancer Genome Atlas (TCGA) database was used to analyze the association between RELA expression levels and miR-21 expression levels, as well as the association between miR-21 expression levels and ARHGAP24 expression levels in patients with PDAC. The F-test was used to determine the significance of the association. The Gene Expression Profiling Interactive Analysis (GEPIA2) and the Human Protein Atlas (HPA) databases were used to detect the expression of RELA in patients with PDAC 14-16. The University of California Santa Cruz Genome Browser and JASPAR 17 were used to predict transcription factors that may interact with miR-21. TargetScan 18, PicTar and miRanda were used to predict potential mRNAs that may be targeted by miR-21. Oncomine gene expression array datasets (https://www.oncomine.org/resource; an online cancer microarray database) 19 were used to analyze the transcription levels of ARHGAP24 in PDAC. The mRNA expression levels of ARHGAP24 in clinical PDAC specimens were compared with those in normal control specimens.

### Cell lines and culture

The human pancreatic cancer PANC-1 and MIA PACA-2 were obtained from the Type Culture Collection of the Chinese Academy of Science (Shanghai, China). Cell lines were cultured in high-glucose DMEM (Gibco; Thermo Fisher Scientific, Inc.) supplemented with 10% FBS (Gibco; Thermo Fisher Scientific, Inc.). Cells were incubated in a humidified incubator at 37 °C with 5% CO_2_.

### Tissue microarray (TMA)

The clinical characteristics of miR-21 and ARHGAP24 expression in PDAC patients were analyzed using TMAs obtained from 69 patients who were diagnosed with PDAC. The use of clinical samples was approved by the Ethics Committee of Shanghai General Hospital and written informed consent was obtained from all patients.

### Reverse transcription-quantitative PCR (RT-qPCR)

Total RNA was isolated from cells using RNAiso Plus Reagent (Takara Bio, Inc.). RT was performed using the PrimeScript^™^ RT reagent kit (Takara Biotechnology Co., Ltd.) and amplified with SYBR^®^ Premix Ex Taq™ (Takara Biotechnology Co., Ltd.). Expression levels were determined using the 2^-ΔΔCq^ method 20. U6 and GAPDH were used as internal reference genes for miRNA and mRNA, respectively.

### Western blotting

Cells were lysed using RIPA buffer (Beyotime Institute of Biotechnology). Subsequently, total protein was separated using SDS-PAGE and transferred to a PVDF membrane. Membranes were blocked for 1.5 h in 5% fat-free milk at room temperature and were subsequently incubated at 4 °C overnight with the following primary antibodies against: GAPDH (cat. no. 2118, 1:1000; Cell Signaling Technology, Inc.), histone H3 (cat. no. 1791, 1/2000; Abcam), RELA (cat. no. 8242, 1:1000; Cell Signaling Technology, Inc.), ARHGAP24 (cat. no. 203874, 1:800; Abcam), Ki-67 (cat. no. 100130-T32, 1:500; Sino Biological, Inc.), proliferating cell nuclear antigen (PCNA; cat. no. 29, 1:1000; Abcam), Cyclin D1 (cat. no. 134175, 1/10000; Abcam), Bcl2 (cat. no. 182858, 1/2000; Abcam) and Bax (cat. no. 32503, 1/2000; Abcam). All antibodies were used according to the manufacturer's protocol. Following primary incubation, membranes were incubated with secondary antibodies, HRP-conjugated goat anti-rabbit IgG (cat. no. D110058, 1:20000; Sangon Biotech Co., Ltd.) and HRP-conjugated goat anti-mouse IgG (cat. no. D110087, 1:20000; Sangon Biotech Co., Ltd.) at room temperature for 1 h. Proteins were visualized using ECL Reagent (MilliporeSigma).

### Transfection

The miR-21 mimic, miR-21 inhibitor and their respective negative controls were purchased from Guangzhou RiboBio Co., Ltd. The overexpression vector and small interfering RNAs (siRNAs) were synthesized by Guangzhou RiboBio Co., Ltd. Lipofectamine^®^ 3000 (Invitrogen; Thermo Fisher Scientific, Inc.) was used according to the manufacturer's protocol at room temperature for 48 h. The cells were collected for subsequent experiments at 48 h following transfection. The transfection efficiency was determined using RT-qPCR and western blotting. Oligos and primers are presented in Table S1.

### Cell proliferation assay

Cell viability was quantified using the Cell Counting Kit-8 (CCK-8) assay. Cells were seeded into 96-well plates at a density of 3x10^3^ cells/well and were cultured in a humidified atmosphere with 5% CO_2_ at 37 °C. Subsequently, 10 μl CCK-8 reagent was added to each well. The absorbance was measured at 450 nm after 2 h. The CCK-8 assay was performed every 24 hours for 5 consecutive days.

For the EdU cell proliferation assay, cells were plated into 96-well plates at a density of 4.0×10^3^ cells/well and cultured at 37 °C for 48 h. Then cells were incubated with 50 μM EdU solution (Guangzhou RiboBio Co., Ltd.) for 2 h. The wells were washed with PBS, and the cells were fixed with 4% paraformaldehyde (P0099; Beyotime Institute of Biotechnology) at room temperature for 30 min and stained according to the manufacturer's instructions. A fluorescence microscope (Leica Microsystems GmbH) was used to image cells. The number of EdU-positive cells was quantified as follows: (EdU-positive cells/Hoechst-stained cells) ×100%. All experiments were performed in triplicate.

### Flow cytometry

The Annexin V-FITC/PI Apoptosis Detection Kit (Multi Sciences Biotech Co., Ltd.) was used in the present study. Briefly, cells were seeded into 6-well plates and transfected. Cells were harvested and resuspended in 500 ul binding buffer after 48 h. Subsequently, cells were incubated with 5 μl Annexin V-FITC and 10 μl PI at room temperature in the dark for 15 min before analysis via flow cytometry.

For the cell cycle assay, the Cell Cycle Staining Kit (Multi Sciences Biotech Co., Ltd.) was used according to the manufacturer's protocol. Briefly, cells were harvested and fixed in 70% ethanol at 4 °C overnight. Cells were then treated with 100 μl RNase A at 37 °C for 30 min and incubated with 400 μl PI at 4 °C in the dark for 30 min. Cells were then analyzed via flow cytometry.

### Construction of stable knockdown and overexpressed cell lines

The miR-21 overexpression and knockdown lentiviruses and their corresponding controls were purchased from Hanbio Biotechnology Co., Ltd. Cells were seeded into 6-well plates. Once cells reached 50% confluence, they were transduced with the appropriate lentiviruses in the presence of 6 μg/ml polybrene (Hanbio Biotechnology Co., Ltd.). Transduced cells were selected using 2 μg/ml puromycin (Sigma-Aldrich; Merck KGaA) and the transfection efficiency was determined via RT-qPCR.

### Chromatin immunoprecipitation (ChIP) assay

The SimpleChIP Enzymatic ChIP Kit (cat. no. 9003; Cell Signaling Technology, Inc.) was used to perform the ChIP assay according to the manufacturer's protocol. Cells were cross-linked with 1% formaldehyde at room temperature for 10 min. The DNA-protein complexes were immunoprecipitated using RELA antibody (cat. no. 8242; Cell Signaling Technology, Inc.) or rabbit IgG (cat. no. 2729; Cell Signaling Technology, Inc.). The bound DNA fragments were subjected to RT-qPCR and further analyzed via 2% agarose gel electrophoresis.

### Dual-luciferase reporter assay

The Dual-Luciferase Reporter Assay System (Promega Corporation) was used. Luciferase reporter plasmids were synthesized by Guangzhou RiboBio Co., Ltd. For the RELA reporter assay, PDAC cells were co-transfected with luciferase reporter plasmids harboring different predicted RELA binding sites, the pRL-TK vector and RELA overexpression vector or negative control plasmid using Lipofectamine 3000. Subsequently, cells were harvested and lysed after 48 h and luciferase activity was detected using Varioskan Flash (Thermo Fisher Scientific, Inc.).

For the 3'-untranslated region (UTR) reporter assay, PDAC cells were cultured in 24-well plates and were co-transfected with miR-21 mimic, miR-21 inhibitor or their negative controls, and wild-type (Wt-ARHGAP24) or mutant type vector which containing mutations in the predicted miRNA binding sites (Mut-ARHGAP24) using Lipofectamine 3000. After incubation for 48 h, luciferase activity was measured. Luciferase activity was calculated using the ratio of firefly luciferase luminescence to *Renilla* luciferase luminescence and was normalized to control.

### Immunohistochemistry (IHC)

IHC was performed on TMA chips containing 69 pairs of pancreatic cancer samples and xenograft tumor samples. The slides were probed with anti-ARHGAP24 antibody (cat. no. 203874, 1:250; Abcam) overnight. After being incubated with secondary antibody HRP-conjugated goat anti-rabbit IgG (cat. no. GB23303, 1:200; Servicebio Co., Ltd.) at room temperature for 1 h, the samples were analyzed using IHC staining scores based on staining area and staining intensity by three independent observers. IHC staining area was scored as follows: 0 (0-10%); 1 (10-25%); 2 (25-50%); 3(50-75%); and 4 (75-100%). The staining intensity was scored as follows: 0, no staining; 1, weak staining; 2, moderate staining; and 3, strong staining. The final score = IHC staining area score x IHC staining intensity score. A total score ≤4 indicated low protein expression levels. Scores >4 indicated high protein expression levels.

### *In situ* hybridization (ISH)

The expression of miR-21 in the TMA was evaluated using a specific digoxin-labeled miR-21 probe. The TMA was treated with proteinase K and incubated at 37 °C for 10 min following deparaffinization and then with hybridization mix at 60 °C overnight. The TMA were subsequently blocked for 30 min. Next, the TMA was incubated with anti-digoxin labeled alkaline phosphatase for 1 h at 37 °C. After being washed and treated with BCIP/NBT reagent, the TMA samples were mounted with coverslips. ISH staining area was scored as follows: 0 (0-10%); 1 (10-25%); 2 (25-50%); 3 (50-75%); and 4 (75-100%). Staining intensity was determined as follows: 0, no staining; 1, weak staining; 2, moderate staining; and 3, strong staining. The final score = ISH staining area score × ISH staining intensity score.

### Animal models

A xenograft tumor model was used to assess the effect of miR-21 *in vivo*. The animals were housed in individually ventilated cages under specific pathogen‑free conditions under controlled environmental conditions (12 h dark/light cycle; 20‑24 °C; humidity, 55±5%) and were supplied with food and water *ad libitum*. Briefly, 4-week-old male BALB/c nude mice were randomly divided into four groups (n=5). Subsequently, 5×10^6^ human pancreatic cancer MIA PaCa-2 cells [lentiviral vectors of miR-21 overexpression (LV-miR-21) and its negative control (LV-NC), lentiviral vectors of miR-21 sponge (SP-miR-21) and its negative control (SP-NC)] in 150 μl PBS were injected subcutaneously into the right armpit of each mouse. The tumor volume was assessed every 3 days following the initial injection over a week. Tumor volume was calculated using the following formula: volume = length x width^2^ x 0.5. Approximately 3 weeks later, when the maximum diameter of the tumor approached 15 mm, all mice were intraperitoneally injected with 15 mg/kg D-luciferin (Promega Corporation) and the tumor size of these mice was imaged using the Xenogen IVIS Illumina System (Caliper Life Sciences, Inc.). Animals were sacrificed using CO_2_. They were placed in a box and 100% CO_2_ was pumped into the box at a replacement rate of 30% volume/min. After 5 min, death was verified by the lack of movement and breathing, and dilated pupils. Subsequently, all animals were removed and observed for 5 min to further verify death. Tumors were surgically dissected for analysis. In the present study, the largest tumor diameter was observed <1.5 cm. All animal experiments were approved by the Animal Care Committee of Shanghai General Hospital (Shanghai, China).

### Statistical analysis

All statistical analysis was performed using SPSS version 20.0 (IBM Corp.) or GraphPad Prism 7.0 (GraphPad Software, Inc.). Statistical significance between two groups was analyzed with the Student's t‑test. The significance among multiple groups was using one-way ANOVA followed by Tukey's post hoc test. Pearson correlation coefficient (r value) was calculated assuming linear relationship between variables. Student's t-test, Pearson χ^2^ test, or the Mann-Whitney U test were used to statistically analyze the clinicopathological features of patients with PDAC. The survival difference was assessed using the Kaplan-Meier method and log-rank test and the survival curve was made using GraphPad Prism 7.0. P<0.05 was considered to indicate a statistically significant difference.

## Results

### miR-21 is highly expressed in PDAC and regulated by RELA

**Figure 1:**
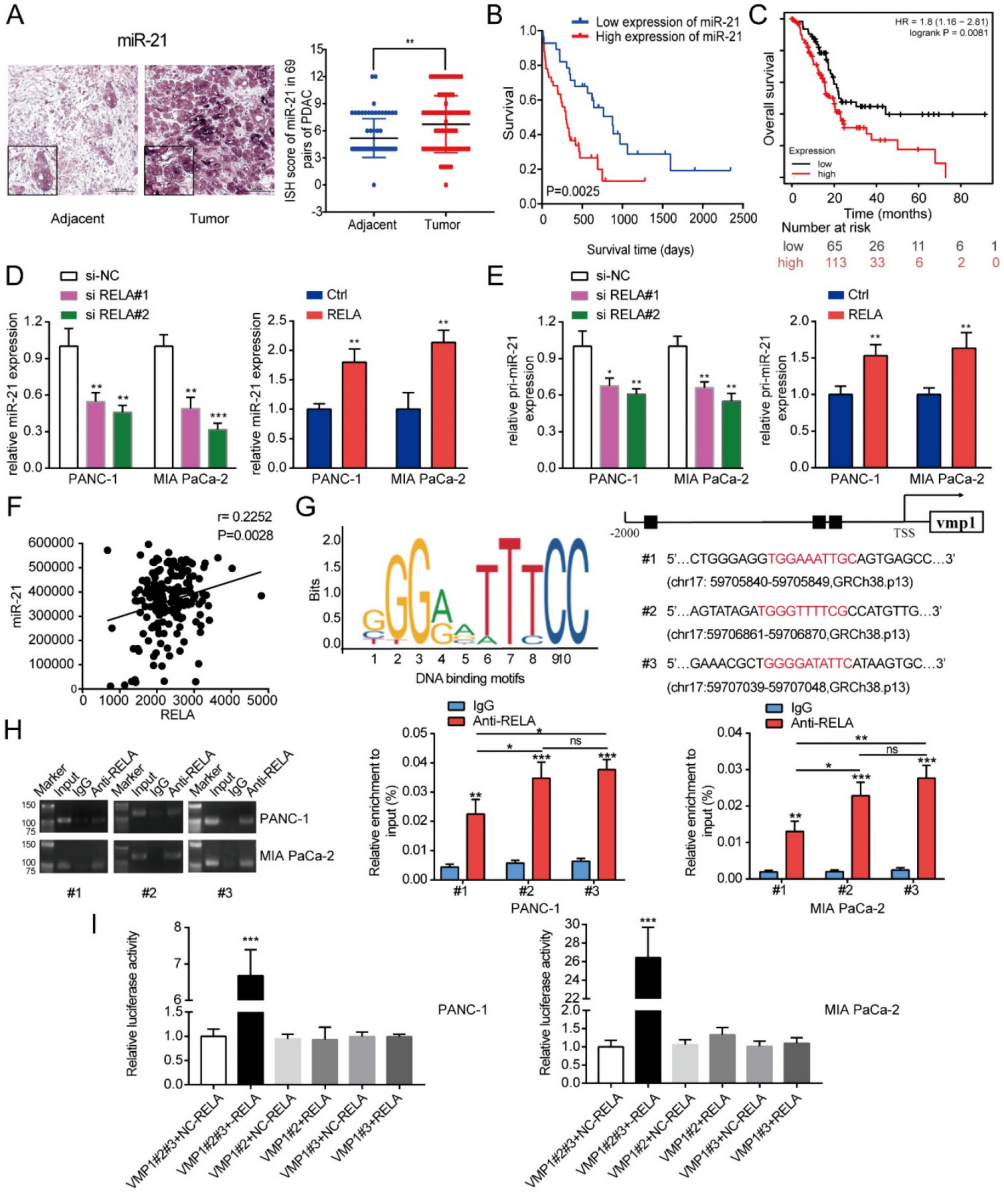
** RELA transcriptionally regulates miR-21 in PDAC cells. (A)** Representative ISH images of miR-21 in the TMA containing 69 pairs of PDAC tissues and adjacent normal tissues (scale bar, 100 µm; magnification, x200). **(B)** Overall survival of patients with low and high miR-21 expression levels was analyzed via ISH of the TMA. **(C)** Kaplan-Meier curves for the overall survival probability in 178 patients with PDAC with low (n=65) and high (n=113) miR-21 expression levels. Curves were produced using a Kaplan-Meier plotter and analyzed using the log-rank test. **(D)** Expression levels of miR-21 in PDAC cells following transfection with si-RELA or RELA overexpression plasmid. **(E)** Expression level of pri-miR-21 in PDAC cells after transfection with si-RELA or RELA overexpression plasmid. **(F)** The expression of RELA and miR-21 were positively correlated in PDAC by TCGA database analysis. **(G)** DNA-binding motifs of RELA on the promoter of vacuole membrane protein 1 were predicted using the JASPAR database. **(H)** Chromatin immunoprecipitation-quantitative PCR assays were performed on the RELA promoter using an anti-RELA antibody in PANC-1 and MIA PaCa-2 cells. **(I)** Luciferase activity in PANC-1 and MIA PaCa-2 cells transfected with the pRL-TK vector and RELA overexpression vector or negative control plasmid was detected. All experiments were performed in triplicate. Data are presented as the mean ± SD. ^*^P<0.05, ^**^P<0.01 and ^***^P<0.001.

To investigate the expression of miR-21 in PDAC, a TMA comprising of 69 pairs of pancreatic cancer samples was used in the subsequent experiments. According to the ISH analysis of miR-21 expression in PDAC tissues on TMA, it was demonstrated that miR-21 expression levels were significantly higher in tumor tissues compared with normal adjacent tissues (Fig. 1A). The Kaplan-Meier survival curve based on the TMA staining results demonstrated that miR-21 overexpression was significantly related to poor overall survival in patients with PDAC (Fig. 1B). Moreover, these results were further supported by the Kaplan-Meier plotter database (Fig. 1C).

Subsequently, potential factors that induced the high expression of miR-21 in PDAC were explored. JASPAR 17 was used to identify potential transcription factors that bind to the promoter region of vacuole membrane protein 1 (VMP1; the encoding gene of miR-21). This analysis identified a cluster of oncogenic transcriptional factors, including RELA and STAT3, which bind to the promoter region of VMP1. Moreover, analysis of samples from patients with pancreatic cancer from the TCGA database, confirmed that the expression levels of RELA and miR-21 were positively associated (Fig. 1F). Consistent with this result, we found that RELA is abnormally upregulated in pancreatic cancer cells and positively correlated with the expression level of miR-21 (Fig. S1A). Furthermore, through GEPIA2 and HPA databases, we identified that RELA is elevated in pancreatic cancer tissue (Fig. S1B and C). Therefore, RELA was focused on in the subsequent experiments. RELA was silenced to assess changes in miR-21 expression. The results demonstrated that miR-21 expression levels were decreased. Furthermore, RELA overexpression increased miR-21 expression levels (Fig. 1D). Then, we found that silencing RELA repressed, while RELA upregulation increased, the expression of primary miR-21 (pri-miR-21), indicating that RELA regulated miR-21 expression at the transcriptional level (Fig.1E). RELA expression levels in PDAC cells following transfection with si-RELA or RELA overexpression plasmid were evaluated using RT-qPCR, western blotting and immunofluorescence (Fig. S2A-C). RT-qPCR demonstrated that si-RELA#2 was more efficient than si-RELA#1 and therefore si-RELA#2 was used for the subsequent experiments. Based on the analysis of the genomic signatures of VMP1, RELA motifs were identified and the following three binding sites were determined using the JASPAR database: Chromosome (chr)17: 59705840-59705849, GRCh38.p13 (#1); chr17: 59706861-59706870, GRCh38.p13 (#2); and chr17: 59707039-59707048, GRCh38.p13 (#3) (Fig. 1G). Subsequently, a ChIP assay was performed (Fig. 1H). The results demonstrated that RELA directly bound to all three predicted binding sites, among which regions #2 and #3 proved to bind more securely to the promoter. Therefore, region #2 and #3 were selected for further study via the dual-luciferase reporter assay. The results indicated that the upregulation of RELA activated the luciferase activity of the plasmid containing intact binding sites of regions #2 and #3, whereas there was no activation for the plasmid containing only one binding site (Fig. 1I). Therefore, the results indicated that RELA may bind to regions #2 and #3 of the VMP1 promoter.

### miR-21 upregulation promotes PDAC cell proliferation and tumor growth

**Figure 2:**
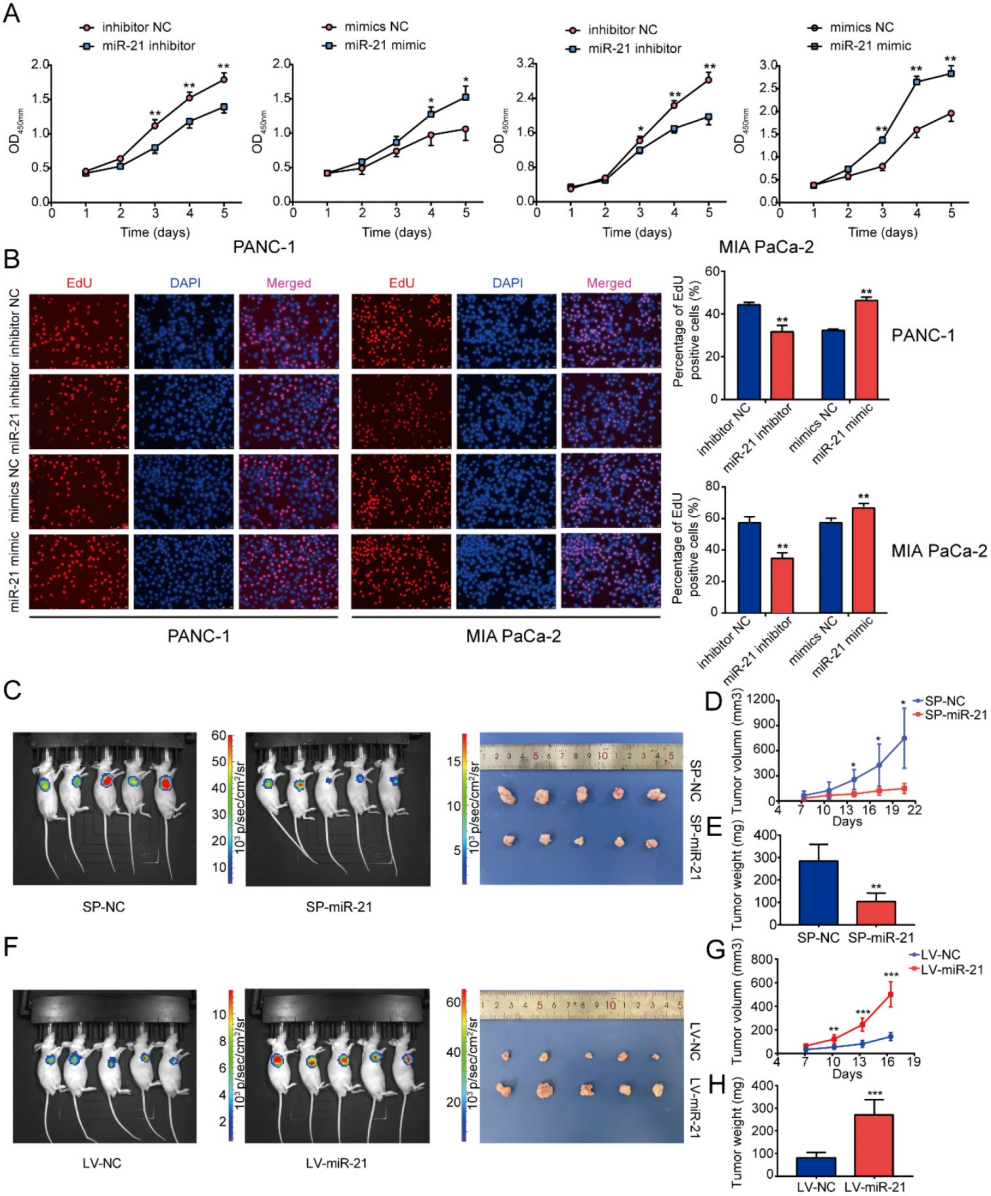
** miR-21 accelerates tumor growth *in vitro* and *in vivo*. (A)** Cell viability assays demonstrated that miR-21 promoted PDAC cell proliferation. **(B)** EdU staining demonstrated that miR-21 promoted PDAC cell proliferation (scale bar, 100 µm; magnification, x200). **(C)** Bioluminescence imaging exhibited the final tumor volume of SP-miR-21 group and SP-NC group. **(D)** Tumor growth in SP-miR-21 group and SP-NC group. **(E)** Quantitative analysis of the final tumor weights in SP-miR-21 group and SP-NC group. **(F)** Bioluminescence imaging exhibited the final tumor volume of LV-miR-21 group and LV-NC group. **(G)** Tumor growth in LV-miR-21 group and LV-NC group. **(H)** Quantitative analysis of the final tumor weights in LV-miR-21 group and LV-NC group. All experiments were performed in triplicate. Data are presented as the mean ± SD. ^*^P<0.05, ^**^P<0.01 and ^***^P<0.001.

To determine the function of miR-21 in PDAC, the miR-21 mimic and miR-21 inhibitor were transfected into PANC-1 cells and MIA PaCa-2 cells. Following transfection, miR-21 expression levels were detected via RT-qPCR (Fig. S2D). PDAC cells exhibited decreased proliferation in the miR-21 inhibitor group, which was determined using cell viability assays and EdU staining (Fig. 2A and B). Furthermore, the miR-21 mimic promoted cell proliferation.

To determine the role of miR-21 *in vivo*, MIA PaCa-2 cells were used to construct stable cell lines. The lentiviral vector for miR-21 overexpression (LV-miR-21), miR-21 sponge (SP-miR-21) and their corresponding NCs (LV-NC and SP-NC, respectively) were used to transduce MIA PaCa-2 cell lines. Transfection efficiency was assessed using RT-qPCR (Fig. S2E). The results of the nude mouse xenograft model indicated that tumors formed by SP-miR-21 cells grew slower than the control group. Conversely, tumors derived from LV-miR-21 cells grew faster when compared with the LV-NC cell group (Fig. 2D and G). Approximately 3 weeks later, it was observed that the luciferase activity of the SP-miR-21 group was lower compared with the SP-NC group, which was determined via bioluminescence imaging.

Moreover, LV-miR-21 mice exhibited higher luciferase activity compared with LV-NC mice (Fig. 2C and F). Subsequently, the nude mice were euthanized and the tumors were removed. The tumors in the SP-miR-21 group were smaller compared with the SP-NC group, whereas tumors in the LV-miR-21 group were significantly larger in volume compared with the LV-NC cell group. Tumors in the SP-miR-21 group were also lighter compared with the SP-NC group (Fig. 2E), whereas the final tumor weight was higher in the LV-miR-21 group (Fig. 2H). These results indicated that miR-21 upregulation may promote PDAC cell proliferation and tumor growth both *in vitro* and *in vivo*.

### miR-21 promotes cell cycle progression and inhibits cell apoptosis in PDAC cells

**Figure 3:**
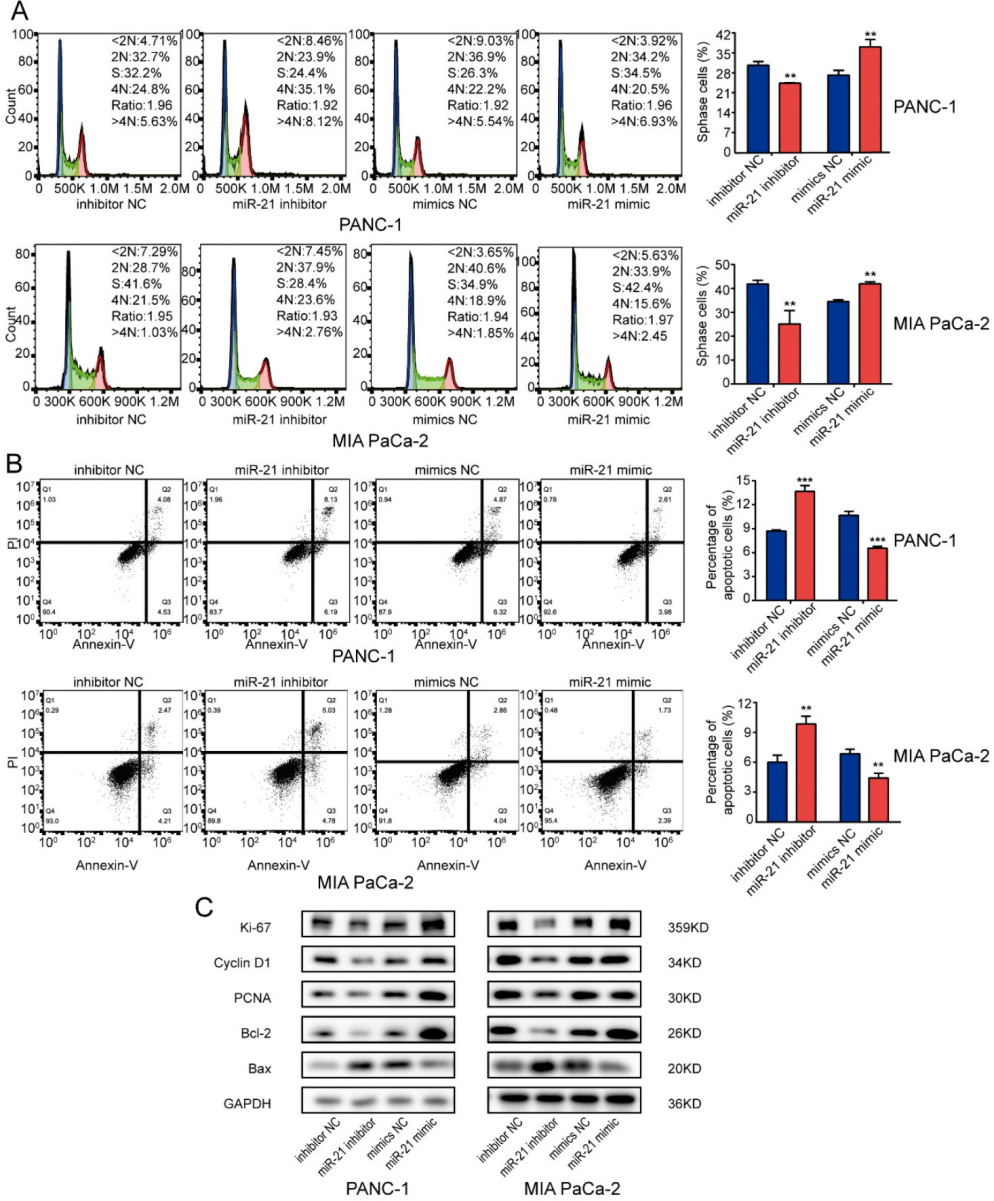
** miR-21 overexpression promotes pancreatic ductal adenocarcinoma cell cycle progression and inhibits apoptosis. (A)** Analysis of the cell cycle was performed using PI staining in PANC-1 cells and MIA PaCa-2 cells transfected with miR-21 inhibitor, miR-21 mimic, or the corresponding control, followed by flow cytometric analysis. **(B)** Cell apoptosis was assessed via FITC-Annexin V and PI staining in PANC-1 cells and MIA PaCa-2 cells transfected with miR-21 inhibitor, miR-21 mimic, or the corresponding control, followed by flow cytometric analysis. **(C)** Expression levels of proliferation markers, cell cycle-related proteins and apoptosis-related proteins in PANC-1 and MIA PaCa-2 cells after transfection with miR-21 inhibitor, miR-21 mimic or the corresponding control, were determined. All experiments were performed in triplicate. Data are presented as the mean ± SD. ^*^P<0.05, ^**^P<0.01 and ^***^P<0.001.

To determine whether miR-21 affected the cell cycle or apoptosis in PDAC cells, cells were transfected with miR-21 mimic and miR-21 inhibitor and any changes in function were assessed using flow cytometry. The results suggested that the miR-21 inhibitor repressed cell cycle progression. However, miR-21 overexpression facilitated PDAC cell cycle progression but suppressed cell apoptosis. Silencing miR-21 had the opposite effect (Fig. 3A and B).

Subsequently, the expression levels of several key regulators involved in cell proliferation, the cell cycle and apoptosis were examined. It was demonstrated that the miR-21 inhibitor decreased the expression levels of the proliferation-associated proteins, Ki-67, Cyclin D1 and PCNA and apoptosis-associated protein Bcl-2, whereas the expression levels of the apoptosis-associated protein Bax were increased. However, the miR-21 mimic had the opposite effect on the expression levels of Ki-67, PCNA, Cyclin D1, Bcl-2 and Bax (Fig. 3C). These results indicated that miR-21 may serve a vital role in cell cycle progression and apoptosis.

### ARHGAP24 is negatively regulated by miR-21 in PDAC

**Figure 4:**
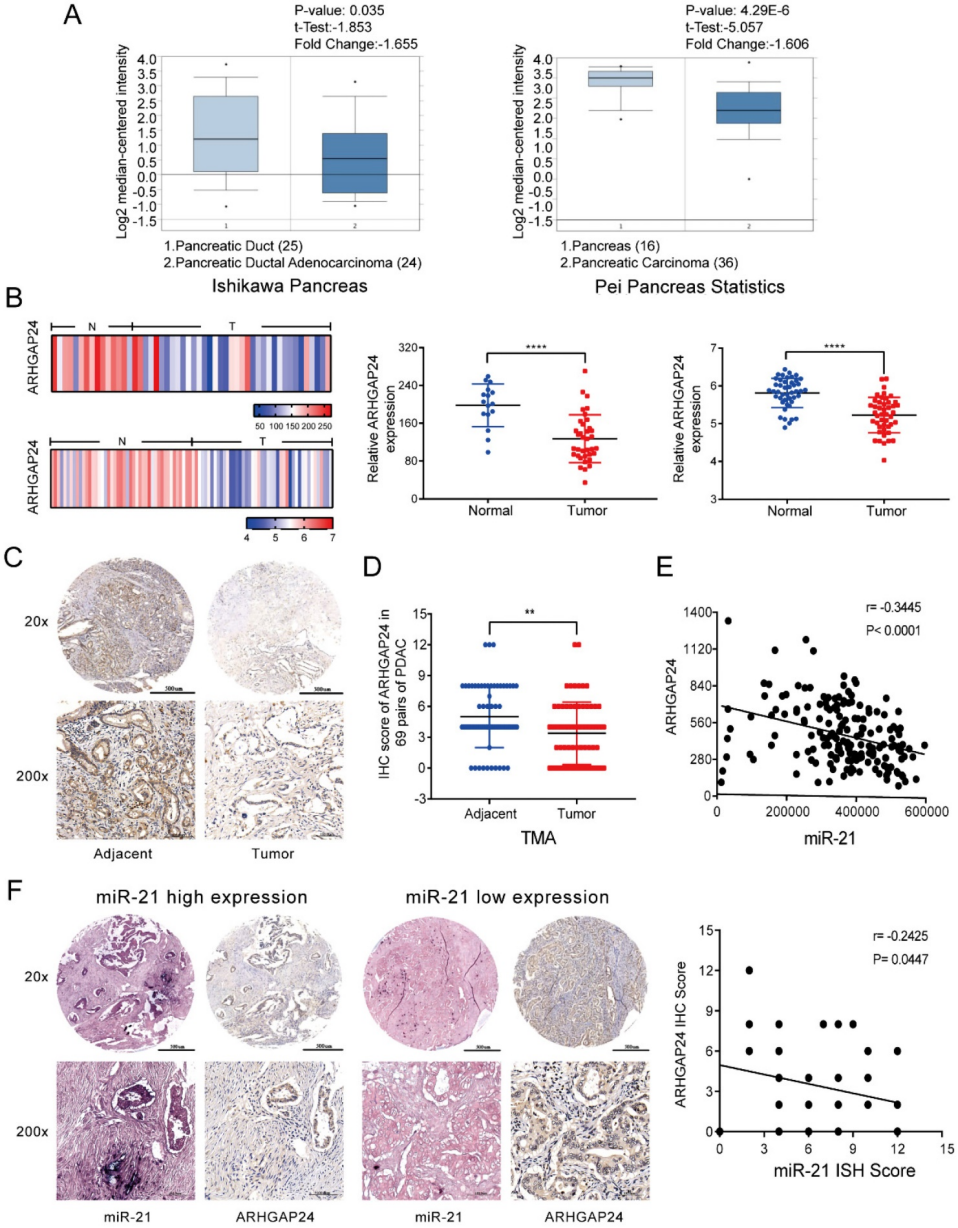
** ARHGAP24 is negatively related to miR-21 in PDAC. (A)** Expression analysis of ARHGAP24 in the Oncomine database. **(B)** ARHGAP24 mRNA expression levels in Gene Expression Omnibus datasets, GSE16515 (N, n=16; T, n=36) and GSE28735 (N, n=45; T, n=45). **(C)** Representative IHC images of ARHGAP24 in the TMA (top image scale bar, 500 µm; top image magnification, x20; bottom image scale bar, 100 µm; bottom image magnification, x200). **(D)** IHC scores for ARHGAP24 protein expression in the TMA. **(E)** ARHGAP24 expression is negatively related to miR-21 expression, which was determined using The Cancer Genome Atlas. **(F)** Representative IHC images of miR-21 and ARHGAP24 expression levels in miR-21 high expression or low expression tissues (top image scale bar, 500 µm; top image magnification, x20; bottom image scale bar, 100 µm; bottom image magnification, x200). ^*^P<0.05, ^**^P<0.01, ^***^P<0.001 and ^****^P<0.0001.

To understand the mechanism by which miR-21 exerts its function in PDAC, miRNA target prediction algorithms, including TargetScan, PicTar and miRanda, were used to identify potential targets of miR-21. Based on a combination of the literature and preliminary experiments, focus was given to ARHGAP24, which acts as a tumor suppressor gene in numerous types of cancer 12, 21. Using the Oncomine database the results demonstrated that the mRNA expression levels of ARHGAP24 are downregulated in pancreatic cancer (Fig. 4A). These findings were confirmed using the NCBI Gene Expression Omnibus datasets (GSE16515, P<0.0001; GSE28735, P<0.0001) (Fig. 4B). Subsequently, ARHGAP24 protein expression levels were determined via analysis of the TMA via IHC. It was observed that ARHGAP24 protein expression levels were downregulated in PDAC samples (Fig. 4C and D). Analysis using TCGA also demonstrated that ARHGAP24 expression was negatively related to miR-21 (Fig. 4E). ISH and IHC scores indicated that there was a negative association between miR-21 and ARHGAP24 expression levels in PDAC samples (Fig. 4F). These results indicated that ARHGAP24 expression levels were negatively regulated by miR-21.

### ARHGAP24 is a direct target of miR-21 in PDAC

**Figure 5:**
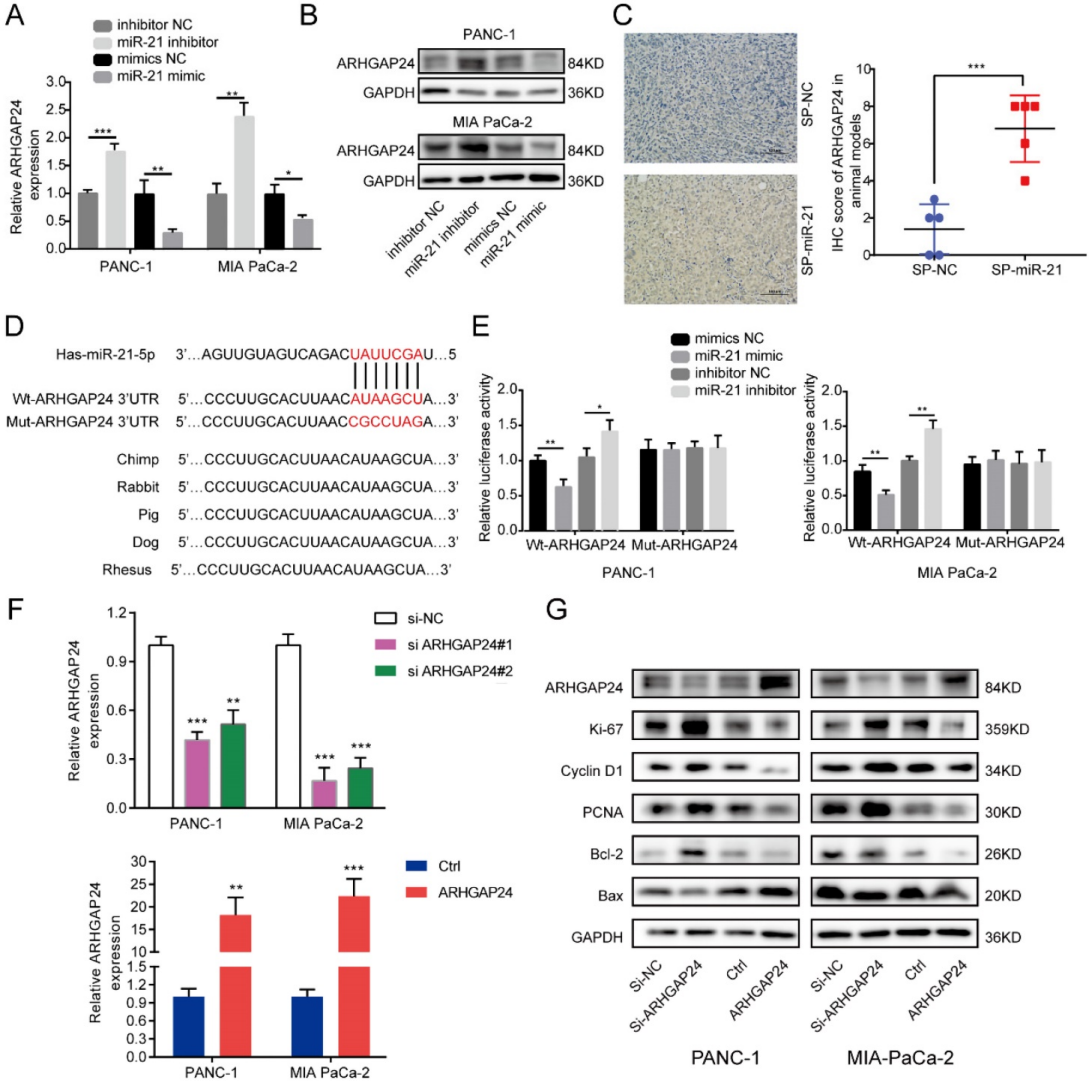
** ARHGAP24 is a direct target of miR-21 in pancreatic ductal adenocarcinoma. (A and B)** RT-qPCR and western blotting were used to analyze cells transfected with the miR-21 inhibitor, miR-21 mimic or the corresponding controls. **(C)** Immunohistochemistry was used to assess xenograft tumors following transfection with SP-NC or SP-miR-21 MIA PaCa-2 cells (scale bar, 100 µm; image magnification, x200). **(D)** Predicted interaction between miR-21 and its putative binding sites in the 3'UTR of ARHGAP24. **(E)** Luciferase activity of ARHGAP24 wild-type and mutated-type transfected with the miR-21 mimic or miR-21 inhibitor. **(F)** Expression level of ARHGAP24 mRNA after transfected with si-ARHGAP24 or ARHGAP24 plasmid was evaluated by qRT-PCR. **(G)** Expression levels of ARHGAP24, proliferation markers, cell cycle-related proteins and apoptosis-related proteins in PANC-1 and MIA PaCa-2 cells following transfection with si-ARHGAP24 and the ARHGAP24 plasmid, or the control. All experiments were performed in triplicate. Data are presented as the mean ± SD. ^*^P<0.05, ^**^P<0.01 and ^***^P<0.001.

To clarify the regulatory relationship between miR-21 and ARHGAP24 in PDAC, cells were transfected with the miR-21 mimic, miR-21 inhibitor and their corresponding controls. miR-21-overexpressing cells exhibited lower ARHGAP24 expression levels, whereas PDAC cells transfected with the miR-21 inhibitor exhibited higher ARHGAP24 expression levels (Fig. 5A and B). Furthermore, the IHC results of the xenograft tumors demonstrated that the SP-miR-21 group exhibited significantly higher ARHGAP24 protein expression levels compared with the SP-NC group (Fig. 5C).

To further investigate if miR-21 interacted directly with the 3'UTR of ARHGAP24, bioinformatics analysis was used to identify potential binding sites, and the dual-luciferase reporter assay was performed to verify this analysis. The results demonstrated that luciferase activity was significantly decreased after PDAC cells were co-transfected with the wild-type ARHGAP24 3'UTR construct and miR-21 mimic, whereas the luciferase activity was increased when cells were treated with the wild-type ARHGAP24 3'UTR construct and miR-21 inhibitor. However, neither miR-21 mimic nor miR-21 inhibitor affected the luciferase activity of PDAC cells transfected with the mutant ARHGAP24 3'UTR (Fig. 5D and E). The results therefore demonstrated that ARHGAP24 may be a direct target of miR-21 in PDAC.

### ARHGAP24 suppresses PDAC growth *in vitro*

**Figure 6:**
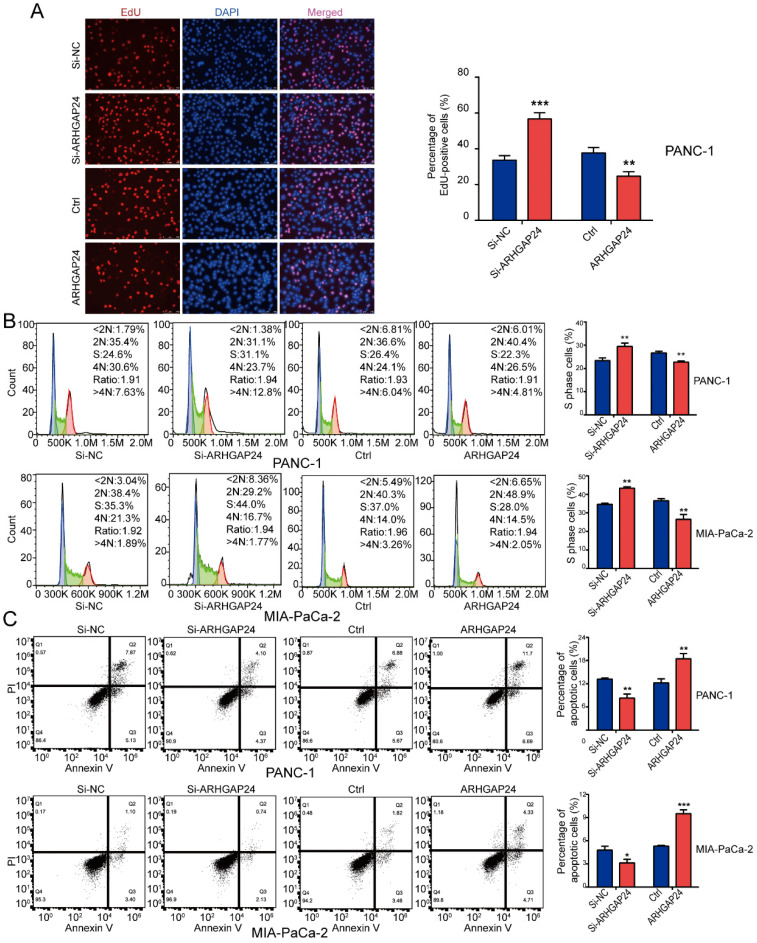
** ARHGAP24 suppresses cell proliferation and the cell cycle, and promotes apoptosis in pancreatic ductal adenocarcinoma. (A)** EdU assays demonstrated that ARHGAP24 suppressed PANC-1 cell proliferation. **(B)** PI staining, followed by flow cytometric analysis, was used to analyze the cell cycle in PANC-1 and MIA PaCa-2 cells transfected with si-ARHGAP24 and the ARHGAP24 plasmid or the control. **(C)** Cell apoptosis was assessed using FITC-Annexin V and PI staining, followed by flow cytometric analysis, in PANC-1 cells and MIA PaCa-2 cells transfected with si-ARHGAP24 and ARHGAP24 plasmid or the control. All experiments were performed in triplicate. Data are presented as the mean ± SD. ^*^P<0.05, ^**^P<0.01 and ^***^P<0.001.

To determine the functional role of ARHGAP24 in PDAC cells, siRNAs and an overexpression plasmid were constructed targeting ARHGAP24. Following transfection, ARHGAP24 mRNA and protein expression levels were detected using RT-qPCR and western blotting, respectively (Fig. 5F and G). The RT-qPCR results exhibited that si-ARHGAP24#1 was more efficient than si-ARHGAP24#2 and therefore si-ARHGAP24#1 was selected for use in subsequent experiments. The results demonstrated that silencing ARHGAP24 promoted the proliferation of PANC-1 and MIA PaCa-2 cells, which was determined using cell viability and EdU assays. However, ARHGAP24 overexpression was demonstrated to exhibit the opposite effect on cell proliferation (Fig. 6A and S3A and B). In terms of the effect of ARHGAP24 on the cell cycle and apoptosis, ARHGAP24 overexpression inhibited cell cycle progression and promoted apoptotic progression. However, si-ARHGAP24 had the opposite effect on the cell cycle and apoptosis progression (Fig. 6B and C). Western blotting demonstrated that si-ARHGAP24 increased Ki-67, PCNA, Cyclin D1 and Bcl-2 protein expression levels, but decreased those of Bax. ARHGAP24 overexpression had an opposite effect. These results indicated that ARHGAP24 may act as a tumor suppressor in PDAC cells.

### ARHGAP24 mediates the effects of miR-21 in PDAC cells

**Figure 7:**
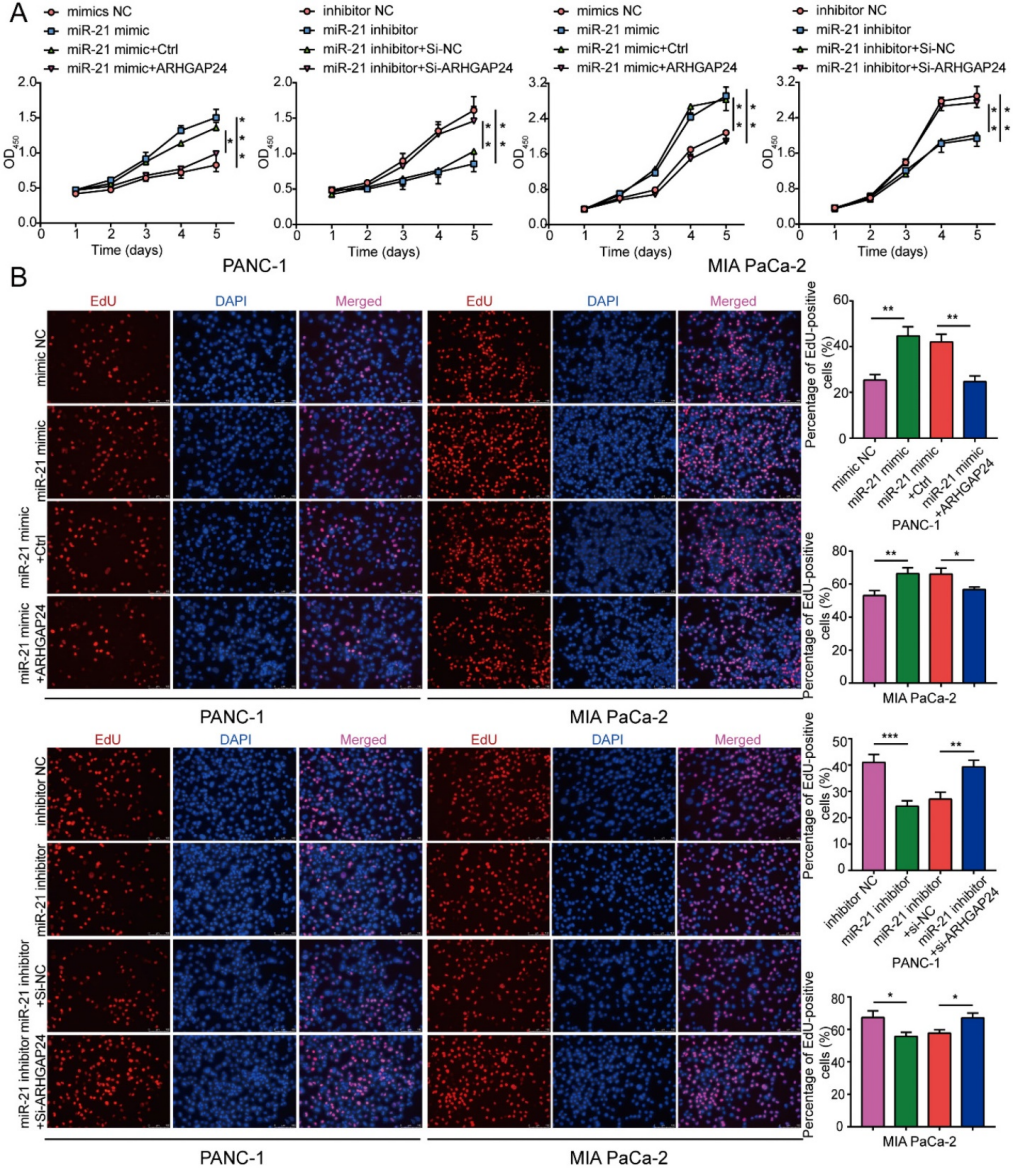
** ARHGAP24 mediates the function of miR-21 in PDAC cell proliferation. (A)** Cell viability assays were used to confirm the proliferative activity of PANC-1 and MIA PaCa-2 cells. **(B)** EdU assays were used to confirm the proliferative activity of PANC-1 cells and MIA PaCa-2 cells. All experiments were performed in triplicate. Data are presented as the mean ± SD. ^*^P<0.05, ^**^P<0.01 and ^***^P<0.001.

To explore whether ARHGAP24 was a contributor to the malignant phenotypes induced by miR-21, rescue experiments were conducted. The results demonstrated that miR-21 could not promote cell proliferation efficiently when co-transfected with the ARHGAP24 overexpression plasmid. Furthermore, when co-transfected with the miR-21 inhibitor and si-ARHGAP24 the antiproliferative effect of the miR-21 inhibitor was markedly suppressed (Fig. 7A and B). These results therefore indicated that ARHGAP24 may be a potential target of miR-21 and mediates the effects of miR-21 on PDAC cells.

### Clinicopathological features of miR-21 and ARHGAP24 in patients with PDAC

**Figure 8:**
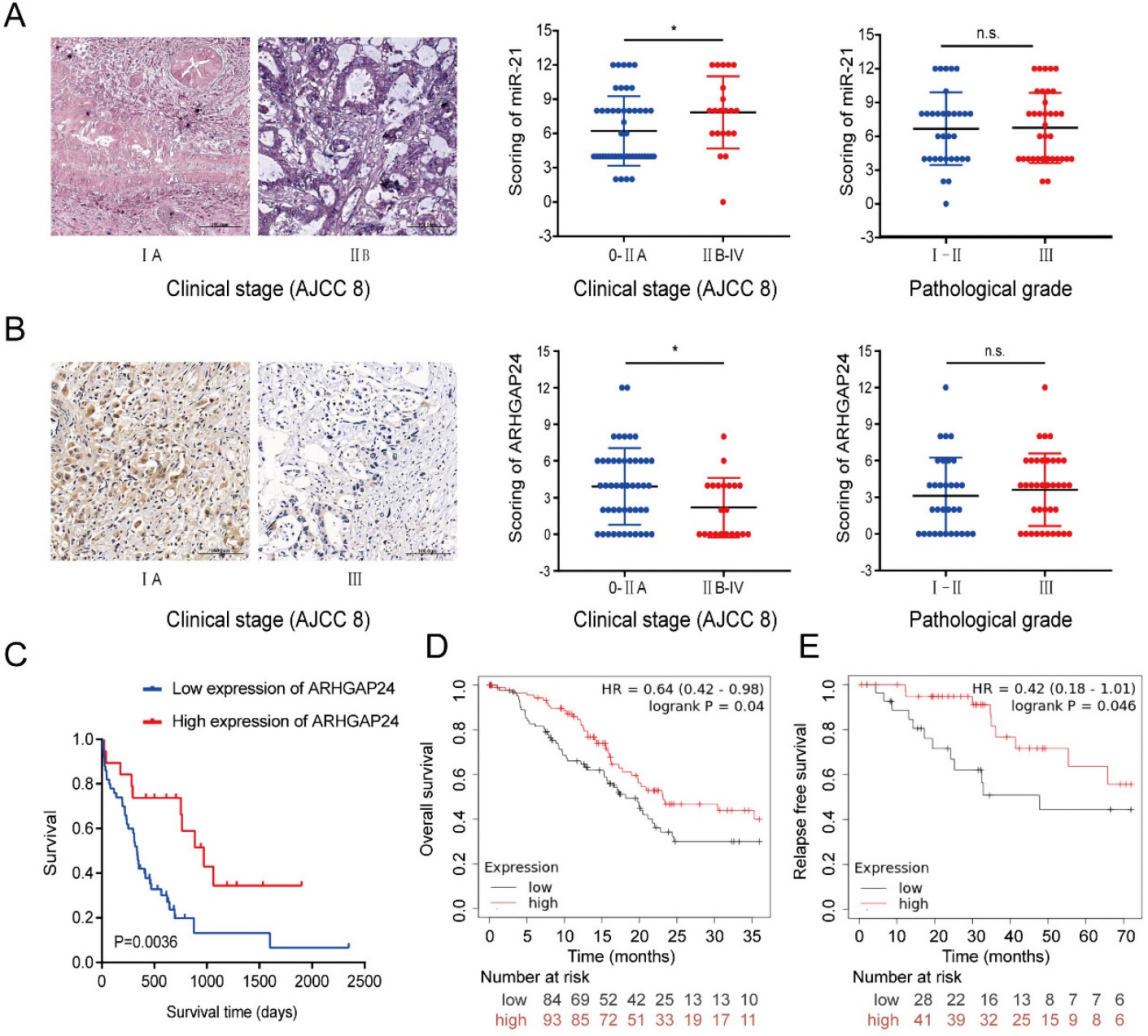
** Clinicopathological features of miR-21 and ARHGAP24 in patients with PDAC. (A)** Representative images of ISH of miR-21 in TMA samples of AJCC 0-IIA vs IIB-IV stage (scale bar, 100 um; magnification, x200). **(B)** Representative images of IHC of ARHGAP24 in TMA samples of AJCC 0-IIA vs IIB-IV stage (scale bar: 100 um, magnification: 200x). **(C)** IHC staining of the TMA determined the overall survival of patients with low and high ARHGAP24 expression levels. **(D)** Overall survival probability in 177 patients with PDAC with low (n=84) and high (n=93) ARHGAP24 expression levels were determined using a Kaplan-Meier plotter and log-rank test. **(E)** Kaplan-Meier curves for relapse free survival in 69 patients with PDAC with low (n=31) and high (n=38) ARHGAP24 expression levels were produced using a Kaplan-Meier plotter, followed by the log-rank test.

A TMA was used to analyze the clinicopathological features of miR-21 and ARHGAP24. The results demonstrated that miR-21 expression levels were significantly higher in PDAC tissues of American Joint Committee on Cancer (AJCC) stage IIB-IV than those of AJCC 0-IIA stages (Fig. 8A). It was subsequently demonstrated that the higher the malignant degree of tumor, the lower the expression of ARHGAP24 (Fig. 8B), and its expression levels were dramatically associated with patient survival. Furthermore, downregulated ARHGAP24 expression levels markedly reduced OS in patients with PDAC (Fig. 8C). Moreover, Kaplan-Meier survival analysis from the Kaplan-Meier Plotter database (http://kmplot.com/analysis/) supported the experimental results, whereby patients with lower ARHGAP24 expression levels had not only a poorer OS but also RFS (Fig. 8D and E). Overall, the results suggested that higher miR-21 expression levels and lower ARHGAP24 expression levels predicted poor survival in patients with PDAC. They therefore have the potential to be prognostic factors of PDAC.

## Discussion

PDAC remains to be an intractable disease due to lack of indicates for early detection, most patients are diagnosed at advanced stage, even in the early stage of disease after surgical resection, lots of patients relapse in 1 year 22. Therefore, the mechanism of the malignant biological feature of PDAC requires further exploration. miR-21 has been reported to be an onco-miR in a number of types of cancer 23-25 and has the potential to be a therapeutic target for cancer treatment. Thus, it is necessary to study the mechanism of miR-21 to accelerate the translation from basic research to clinical application.

In the present study, we found that miR-21 was upregulated in PDAC tissues and its high expression predicted a poor prognosis, current studies mostly focus on the ceRNA mechanism to regulate miRNAs expression, but there are few studies on the transcriptional regulation of miRNAs. In this study, RELA, an oncogenic transcription factor, was demonstrated to activate miR-21 transcription in PDAC. The study in glioma also corroborates with our findings in that RELA promotes the transcription of miR-21 by binding to its promoter 26. Uncontrolled inflammation is a key feature of PDAC and contributes to the activation of the transcription factor NF-κB. Virchow proposed that inflammation and chronic irritation gave rise to cancer in 1863. Subsequently, NF-κB has been confirmed to be involved in numerous types of cancer over the past decades 27-31. RELA, one of the components of the NF-κB signaling pathway, modulates various cell biological functions in pancreatic cancer 32-34. Numerous studies have revealed that certain important genes, such as wild-type p53-induced phosphatase 1, are targets of NF-κB 35. Moreover, non-coding RNA may be activated by NF-κB. For example, RELA binds to the promoter region of nuclear paraspeckle assembly transcript 1 to regulate its expression, which therefore modulates pancreatic cancer cell proliferation and migration 36. miR-21 is repressed by Kruppel Like Factor 9 via binding to gene promoter regions in glioma 37. In this present study miR-21 was identified to be transcriptionally activated by RELA. Transcription factor (TF)-binding sites are small, typically 6-12 bp, and therefore human genes usually contain multiple potential binding sites. The present study demonstrated that RELA bound to the VMP1 promoter in the chr17: 59706861-59706870, GRCh38.p13 and chr17: 59707039-59707048, GRCh38.p13 regions, to facilitate the transcription of VMP1. This may be due to RELA being activated by binding to two binding sites or co-binding with two or more TFs on promoters to recruit a common cofactor 38, 39.

Functional experiments determined that miR-21 could promote PDAC progression both *in vitro* and *in vivo*. Mechanistically, miR-21 downregulated ARHGAP24 expression to eliminate its anti-tumor effect. MiRNAs exert their biological functions via interacting with 3'UTRs of mRNAs 40-43. Bioinformatic tools were used to predict possible targets of miR-21, identifying ARHGAP24. ARHGAP24 belongs to the Rho GTPase activating protein (RHOGAPs) family, which is involved in numerous cell processes. For example, previous studies have reported that ARHGAP24 suppresses cell invasion in triple-negative breast cancer. Moreover, ARHGAP24 affects the STAT3 signaling pathway, which modulates the anti-cancer activity of sorafenib against breast cancer. ARHGAP24 also regulates the cell apoptosis and invasion of renal cell carcinoma 44. The occurrence and development of PDAC is a complex biological process, including the synergy of many factors and many genes, which goes through many complex biological development stages, and is also a manifestation of the imbalance between oncogenes and tumor suppressor genes. There is no research on the role of ARHGAP24 protein in pancreatic cancer. Here, we determined that ARHGAP24 is a direct target of miR-21 and could be negatively regulated by miR-21 in PDAC. ARHGAP24 overexpression suppressed cell proliferation and cell cycle progression and induced cell apoptosis in PDAC cells. Furthermore, it was also demonstrated that ARHGAP24 expression levels were downregulated in PDAC tissues and were negatively correlated with miR-21 expression levels.

In conclusion, the RELA/miR-21/ARHGAP24 axis is important in PDAC progression. In the present study, miR-21 was upregulated and acted as an onco-miR in PDAC, whereby its high expression was a result of RELA activation. Furthermore, ARHGAP24 was a target of miR-21 and acted as a tumor suppressor in PDAC. Clinically, its low expression was associated with a poor prognosis in pancreatic cancer. The present study indicated that both miR-21 and its target ARHGAP24 may be promising prognostic biomarkers and therapeutic targets for PDAC.

## Supplementary Material

Supplementary figures and table.
