# Supplementary material for: RELA-induced MiR-21 Exerts Oncogenic Effects on PDAC via Targeting of ARHGAP24
Source: J Cancer. 2022 Jun 21;13(9):2781–97. doi: 10.7150/jca.73690 (PMC9254878; doi:10.7150/jca.73690)
Supplement: Supplementary file 1 — Supplementary figures and table. [file jcav13p2781s1.pdf]

Supplementary figures

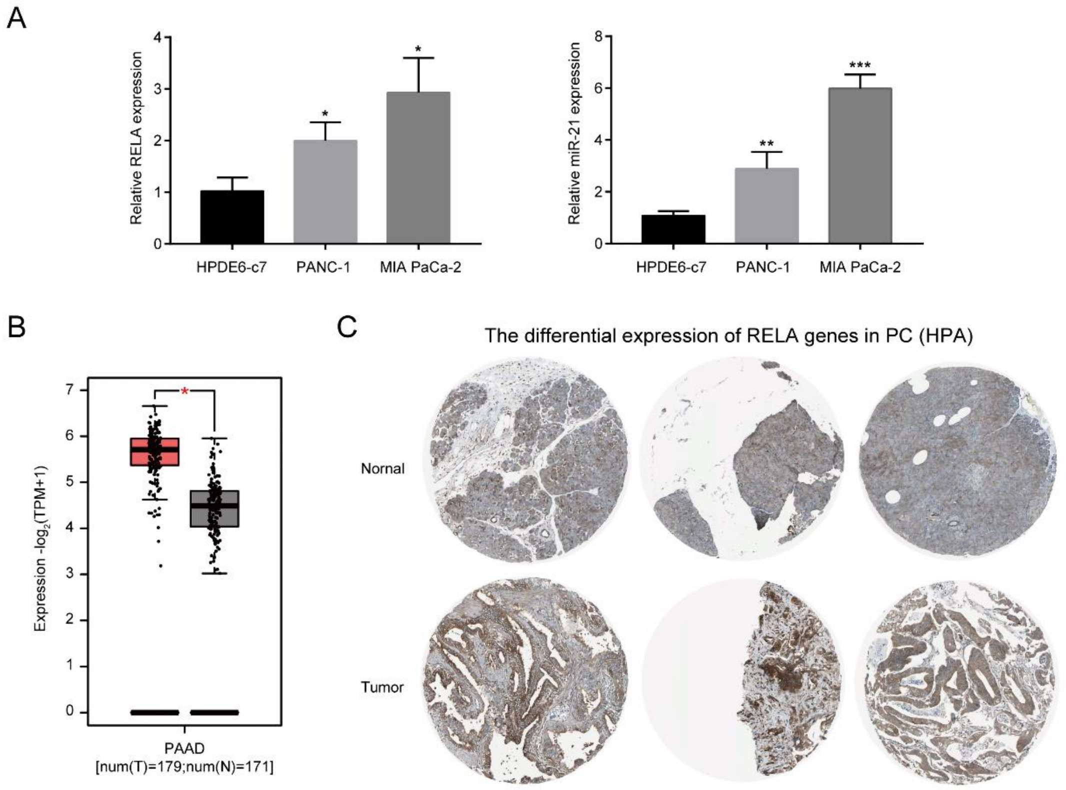

Figure S1. Expression levels of RELA in pancreatic cancer. (A) Reverse transcription-quantitative PCR was used to determine RELA and miR-21 expression levels in pancreatic cancer cells and normal pancreatic ductal epithelial cell (HPDE6-c7). (B) and (C) The expression of RELA in pancreatic cancer tissue through analysis of GEPIA2 and HPA databases. All experiments were performed in triplicate. Data are presented as the mean ± SD. $^{*}P<0.05$, $^{**}P<0.01$ and $^{***}P<0.001$.

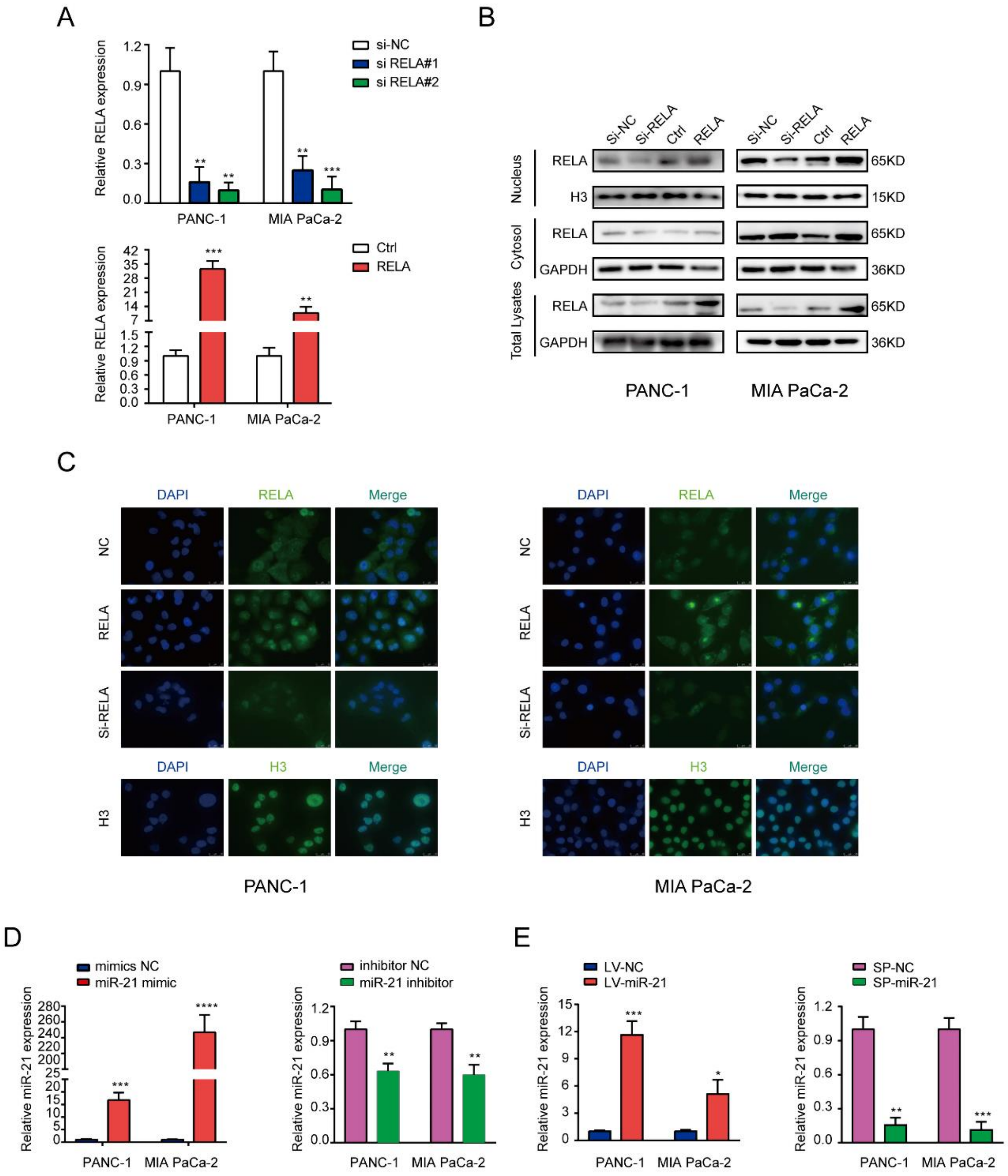

Figure S2. Expression levels of RELA or miR-21 in PDAC cells following transfection. (A) Reverse transcription-quantitative PCR was used to determine RELA mRNA expression levels following transfection with si-RELA or RELA. (B) Western blotting was used to determine the RELA protein expression level following transfection with

si-RELA or RELA. (C) Representative fluorescence images of RELA protein expression levels in PDAC cells following transfection with si-RELA or RELA (scale bar, 25 μm; magnification, x630). (D) miR-21 expression levels in PDAC cells following transfection with miR-21 mimic or miR-21 inhibitor. (E) miR-21 expression levels in PDAC cells following transduction with lentivirus-miR-21 or sponge-miR-21. All experiments were performed in triplicate. Data are presented as the mean ± SD. *P<0.05, **P<0.01 and ***P<0.001.

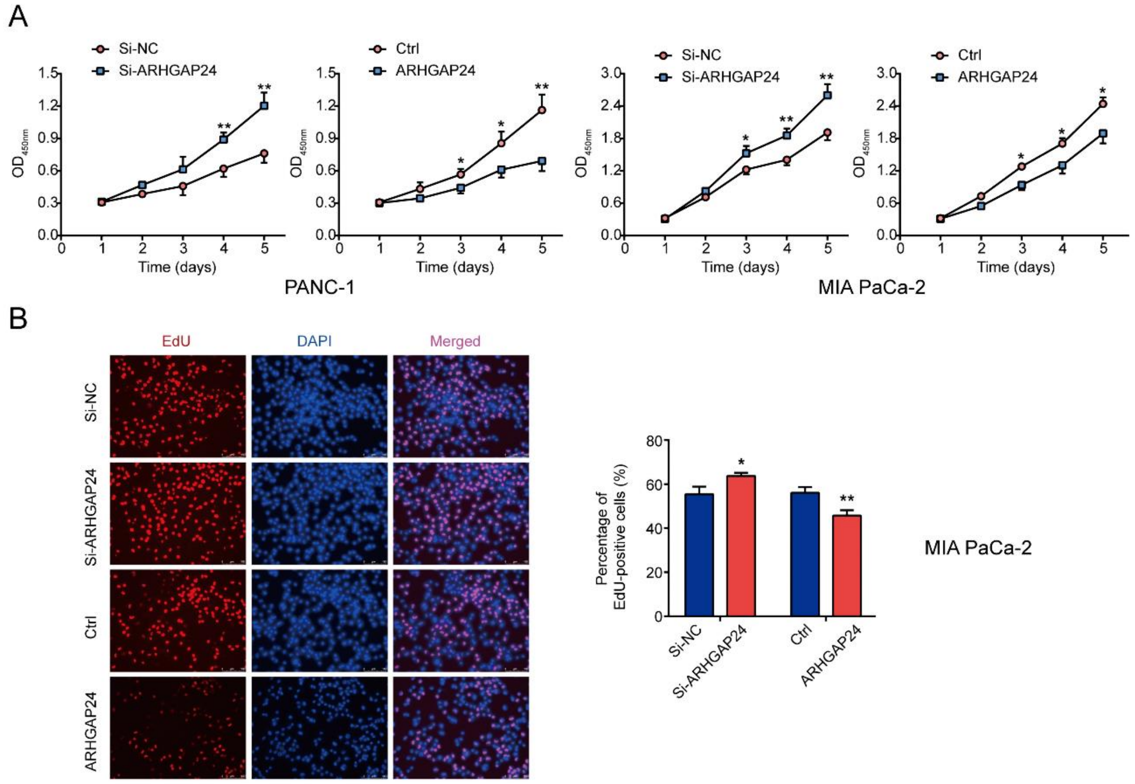

Figure S3. ARHGAP24 promotes cell growth in pancreatic ductal adenocarcinoma. (A and B) Cell viability and EdU assays were performed to measure cell proliferation (scale bar, 100 μm; magnification, x200). All experiments were performed in triplicate. Data are presented as the mean ± SD. *P<0.05 and **P<0.01.

Supplementary Table S1. Oligos and primers used in this study.

Oligonucleotides	Supplier	Catalog number or sequence
Si-h-RELA_001	RiboBio	5’-GCTGCAGTTTGATGATGAA-3
Si-h-RELA_002	RiboBio	5'-CTTCCAAGTTCCTATAGAA-3
GenOFFTM st-h-ARHGAP24_001	RiboBio	5'-GGAGGATACTGTTCGTTAT-3
GenOFFTM st-h-ARHGAP24_002	RiboBio	5'-GAAGGTATCTGGTACCAAA-3
siR NC #1	RiboBio	5'-GGCTCTAGAAAAGCCTATGC-3
RELA in pcDNA3.1-C-3*Flag	RiboBio	Cat# ST001295
ARHGAP24 in pcDNA3.1-C-3*Flag	RiboBio	Cat# ST001274
PcDNA3.1	RiboBio	Cat# ST00001
micrON hsa-miR-21-5p mimic	RiboBio	Sense: UAGCUUAUCAGACUGAUGUUGA<br>Antisense: UCAACAUCAGUCUGAUAAGCUA
micrON mimic NC #22	RiboBio	Sense: UUUGUACUACACAAAAGUACUG<br>Antisense: CAGUACUUUUGUGUAGUACAAA
micrOFF hsa-miR-21-5p inhibitor	RiboBio	UCAACAUCAGUCUGAUAAGCUA
micrOFF inhibitor NC #22	RiboBio	CAGUACUUUUGUGUAGUACAAA
ARHGAP24-3’UTR WT pmirGLO Amp	RiboBio	Cat# ST008732
ARHGAP24-3’UTR MUT pmirGLO Amp	RiboBio	Cat# ST008733
VIMP1 promoter luc pGL3-Basic-3 Amp	RiboBio	Cat# ST009833
VIMP1 promoter luc pGL3-Basic-1 Amp	RiboBio	Cat# ST009831
VIMP1 promoter luc pGL3-Basic-2 Amp	RiboBio	Cat# ST009832
PRL-TK	RiboBio	Cat# ST00009
has-miR-21 reverse transcription primer	EnzyArtisan	5’-GTCGTATCCAGTGCAGGGTCCGAGGTATTCGCACTGGATACGACTCAACA-3’
hsa-U6 reverse transcription	EnzyArtisan	5'-CTCAACTGGTGTCGTGGAGTCGGCAATTC

**primer**		AGTTGAGAAAAATAT -3'
**hsa-U6 forward primer for qPCR**	EnzyArtisan	5'- CAAGGATGACACGCAAA -3'
**hsa-U6 reverse primer for qPCR**	EnzyArtisan	5'- TCAACTGGTGTCGTGG -3'
**GAPDH forward primer for qPCR**	EnzyArtisan	5'-GTCTCCTCTGACTTCAACAGCG -3'
**GAPDH reverse primer for qPCR**	EnzyArtisan	5'-ACCACCCTGTTGCTGTAGCCAA-3'
**RELA forward primer for qPCR**	EnzyArtisan	5'-TGAACCGAAACTCTGGCAGCTG-3'
**RELA reverse primer for qPCR**	EnzyArtisan	5'-CATCAGCTTGCGAAAAGGAGCC-3'
**ARHGAP24 forward primer for qPCR**	EnzyArtisan	5'-ACCTCCGAGAACTTCCAGAACC-3'
**ARHGAP24 reverse primer for qPCR**	EnzyArtisan	5'-CCACTGGCAAACTCTTCACCTG-3'
**miR-21-5p forward primer for qPCR**	EnzyArtisan	5'- CGCGCGTAGCTTATCAGACTGA-3'
**miR-21-5p reverse primer for qPCR**	EnzyArtisan	5'- ATCCAGTGCAGGGTCCGAGG-3'
**Pri-miR-21 forward primer for qPCR**	EnzyArtisan	5'-ACACTCATCCCATTCTAAACTATGTG-3'
**Pri-miR-21 reverse primer for qPCR**	EnzyArtisan	5'-CCAACTACTCTGAAATCTCCATTACTC-3'
**Vmp1#1 forward primer for Chip-qPCR**	EnzyArtisan	5'-TCAGGCTGGGAGGTGGAAA-3'
**Vmp1#1 reverse primer for Chip qPCR**	EnzyArtisan	5'-TGGAGACAGGGTTTTGCATTG-3'
**Vmp1#2 forward primer for Chip-qPCR**	EnzyArtisan	5'-CTGGGATTACAGGCACGCG-3'
**Vmp1#2 reverse primer for Chip qPCR**	EnzyArtisan	5'-AGGCTGATGTGGGCGGATC-3'
**Vmp1#3 forward primer for Chip-qPCR**	EnzyArtisan	5'-GAAACCAATTCTTTCCACGCAG-3'
Vmp1#3 reverse primer for Chip qPCR	EnzyArtisan	5'-ATTGATAAAATAGCGAGCAGGCC-3'
